# Pituitary macroadenomas in childhood and adolescence: a clinical analysis of 7 patients

**DOI:** 10.1186/s40842-023-00153-6

**Published:** 2023-10-31

**Authors:** Cristina Aguilar-Riera, María Clemente, Núria González-Llorens, Eduard Mogas, Ariadna Campos-Martorell, Anna Fàbregas, Betina Biagetti, Elida Vázquez, Diego Yeste

**Affiliations:** 1 Pediatric Endocrinology Service, Hospital Universitari Vall d’Hebron, Barcelona, Spain; 2 CIBER Enfermedades Raras, Instituto Carlos III, Madrid, Spain; 3 Universitat Autònoma de Barcelona, Barcelona, Spain; 4 Endocrinology and Nutrition Service, Hospital Universitari Vall d’Hebron, Barcelona, Spain; 5 Pediatric Radiology Service, Hospital Universitari, Vall d’Hebron, Barcelona, Spain

**Keywords:** Macroadenoma, Child, Adolescent, Cabergoline, Transsphenoidal, Genetic analysis

## Abstract

**Background:**

Pituitary adenomas (PPAs) are uncommon in childhood and adolescence, accounting for 2–6% of all intracranial neoplasms. Delayed puberty, growth retardation, galactorrhea and weight gain are common features at presentation in pediatric patients. Functional tumors constitute a vast majority (90%) of PPAs, with the most frequent being prolactinomas.

**Case presentation:**

A retrospective review of the clinical features and outcomes of 7 pediatric patients with pituitary macroadenomas was conducted. We included PPAs in patients under 18 years at diagnosis with diameters larger than 10 mm by magnetic resonance (MRI).

Six patients were males (85%), with age at diagnosis ranging from 8 to 15 (median 14 ± 2.8SDS). The primary symptoms that led to medical attention were growth retardation, gigantism and secondary amenorrhea. The visual field was reduced in three cases (42%). Suprasellar extension was present in 3 subjects, and one had a giant adenoma. Adenomas were clinically functioning in 6 patients (85%) (three prolactinomas, two somatropinomas, one secreting FSH and one no-producer). The prolactinomas responded to treatment with cabergoline. For the rest, one required transsphenoidal surgery and the other three both surgery and radiotherapy. All patients undergoing radiotherapy had secondary panhypopituitarism. In relation to the genetic studies, two patients presented a pathogenic mutation of the *AIP* gene and one of the *MEN1*.

**Discusion and conclusion:**

Pediatric pituitary macroadenomas are a distinct entity, mostly found in males and with a predominance of functional tumors leading to detrimental effects on growth and puberty in addition to neuro-ophthalmological manifestations. It is important to perform genetic studies in patients with macroadenomas appearing under the age of 18 years as genetic and syndromic associations are more frequent in this age group.

## Background

Pituitary adenomas are rare in childhood and adolescence, representing 2 to 6% of all intracranial neoplasms [1–3]. Most of the available reports are based on individual case studies and only a few series have been reported [4]. Depending on their size, they are classified as microadenomas (< 10 mm), macroadenomas (≥ 10 mm) and giant adenomas (≥ 40 mm) [5–7]. Delayed puberty, growth retardation, galactorrhea, and weight gain are the most common clinical presentations in children [3, 8, 9]. Functioning tumors make up the vast majority (90%) of pituitary adenomas [2, 3, 8, 9], with the prolactin hormone (PRL) tumor prolactinoma, being the most prevalent histological type in children, followed by the adrenocorticotropic hormone (ACTH)-secreting tumor, corticotropinoma, and the growth hormone (GH) secretor somatotropinoma [8, 9]. With reference to the age of presentation, some prolactinoma and somatotropinoma are more prevalent in postpubertal ages, whereas corticotropinoma is more prevalent in the first decade and in prepubertals [2, 8, 10]. Non-functioning pituitary adenomas and those that secrete thyroid-stimulating hormone (TSH) or gonadotropins, follicle-stimulating hormone (FSH), and luteinizing hormone (LH) are rare in children, with a prevalence of only 3–6% of all pituitary tumors [5, 8]. There is a slightly higher prevalence of pituitary adenomas in females, although macroadenomas present more frequently in boys than in girls [8, 9].

Pituitary adenomas in childhood may have a genetic cause and, in some cases, additional manifestations may present as part of a syndromic disease [2, 9, 11–13]. The therapeutic options depend on the type of tumor, with surgery normally being the first option except for prolactinomas [2, 6, 8, 11, 13, 14].

### Patients’ case series report

A retrospective review was conducted of 7 pediatric patients under 18 years of age diagnosed with pituitary macroadenomas equal to or greater than 10 mm in diameter by MRI during the last 10 years in a tertiary hospital. Data were collected covering clinical, biochemical (pituitary hormone basal analysis, no functionally tests were performed), imaging characteristics (MRI latero-lateral diameters), the treatment undertaken and, if applicable, the pathological anatomy, as well as the genetic study (genetic panel Hereditary Plus OncokitDx Healthincore and MiSeq Illumina, bioinformatics SNVs, ins/dels, CNVs, Alus and other by software Data Genomics Imegen; v19.1) and the evolution after treatment (Table 1).

**Table 1:** Clinical, hormonal, and radiological characteristics; treatment; follow-up and results of the genetic study and family history of endocrine neoplasia characteristics of 7 pediatric patients with pituitary macroadenomas

Patient	1	2	3	4	5	6	7
Sex	Male	Female	Male	Male	Male	Male	Male
Age at diagnosis (years)	15	15	14	8	10	14	15
Anthropometry	W: 41.5kg (-2.46SDS) L: 154.2cm (-1.5SDS) IMC: 17.45 kg/m2 (-1.29SDS)	W: 63kg (+ 1.36SDS) L: 152cm (-1.7SDS) IMC: 27.2 kg/m2 (-1.5SDS)	W: 40.7kg (-1.59SDS) L: 151.3cm (-1.68SDS) IMC: 17.8 kg/m2 (-0.92SDS)	W: 36.8kg (-1.63SDS) L: 152.1cm (+ 4.3SDS) IMC: 15.9 kg/m2 (-0.39SDS)	W: 60kg (+ 2.9SDS) L: 160cm (+ 3.3SDS) IMC: 23.4kg/m2 (+ 1.58SDS)	W: 70kg (+ 0.99SDS) L: 180cm (+ 1.8SDS) IMC: 21.6 kg/m2 (-0.09SDS)	W: 60kg (-0.14SDS) L: 160cm (-1.1SDS) IMC: 23.4 kg/m2 (+ 0.57SDS)
Clinical symptoms	Growth retardation, Galactorrhea	Secondary Amenorrhea	Growth retardation	Gigantism	Gigantism	Headache	Polyuria, polydipsia 3L/day
Tanner at diagnosis	P3G3, 12/12cc	S5P5	P3G3, 15/15cc	P1G1, 3/3	P1G1 3/3	G3P3, 15/15	G3P3, 15/15
Visual Defects	Yes	No	No	No	Yes	No	Yes
MRI	Adenoma (15mm)	Adenoma (29mm)	Adenoma (11mm)	Adenoma (16mm)	Adenoma with suprasellar extension (40mm) and cavernous sinus invasion	Adenoma with suprasellar extension (20mm)	Adenoma with suprasellar extension (33mm) and cavernous sinus invasion
Elevated pituitary hormone	Prolactin (583ng/ml)	Prolactin (> 1000ng/ml)	Prolactin (70ng/ml)	IGF1 631ng/ml (+ 3.52 SDS)	IGF1 1115ng/ml (+ 5 SDS)	No	FSH 30U/l (nv: 0.16–3.33 Ui/L)
Adenoma Type	Prolactin secreting PA	Prolactin secreting PA	Prolactin secreting PA	GH-secreting PA	GH-secreting PA	Non-secreting PA	FSH-secreting PA
Other pituitary hormone defects	IGF-I: (154ng/ml (-1.1 SDS) testosterone: 34ng/dl (nv:300 ± 22 ng/dl)	No	IG-FI: 181ng/ml (-0.6 SDS); testosterone: 154ng/ml (nv:300 ± 22 ng/dl)	No	No	No	testosterone: 10ng/dl (nv:300 ± 22 ng/dl), cortisol: < 5ug/dl (nv: 16.3 ± 6ug/dl), TSH < 0.1U/l (nv: 1.9 ± 1.28U/l)
Transsphenoidal surgery	No	No	No	Yes	Yes	Yes	Yes
Pathological anatomy	-	-	-	Hormonal immunohistochemical expression and transcription factors compatible with sparsely granulated somatotrophic adenoma, with a prolactin expression focus	Somatotrophic pituitary adenoma	Adenoma with negative immunoreactivity to all pituitary hormones	Adenoma with immunoreactivity to FSH with a 10–20% cell proliferation index and P53 negative. At reintervention same conclusion
Radiotherapy	No	No	No	No	Yes	Yes	Yes
Medical treatment	Cabergoline 3mg/week	Cabergoline 1mg/week	Cabergoline 1mg/week	No	No	No	No
Complications	No	No	No	ACTH deficiency	Secondary panhypopituitarism	Secondary panhypopituitarism	Secondary panhypopituitarism
Follow up	3 months MRI tumor reduction (19mm) Prolactin 116ng/ml	2 years MRI tumor reduction (4mm) Prolactin 14ng/ml Cabergoline 0.5mg/week	3 months MRI tumor stabilization (7mm) Prolactin 10.9ng/ml Testosterone: 451ng/ml	6 months IGF1 110ng/ml (-0.16DS) MRI secondary changes to surgery	10 years IGF-I: 107ng/ml(-1SDS) MRI tumor remnants persist	10 years MRI tumor remnants persist	10 years MRI tumor remnants persist
Genetics	In progress	*MEN1* Heterozygous change c.249_252delGTCT; p(IIe85Serfs*33	Negative: *NF1, AIP, CDKN1B, CDC73, MEN1, RET, PRKAR1A, BRCA1, BECA2, MLH1, MSH2, MSH6*	*AIP* heterozygous change c.811C > T; pArg271Trp	*AIP* heterozygous change c.811C > T; pArg271Trp	Unrealized	Unrealized
Secondary tumors	No	Parathyroid adenoma	No	No	No	No	No
Family history	No	Sister with parathyroid adenoma and same pathogenic variant	No	Mother has same pathogenic variant without clinical manifestations	Mother and brother have same pathogenic variant without clinical manifestations Maternal aunt and cousin affected by pituitary adenomas with the same pathogenic variant	No	No

We present a total of 7 patients, 6 of whom were male (85%), with an age at diagnosis between 8 and 15 years (14 ± 2.8SDS). In all cases, MRI imaging revealed a pituitary macroadenoma with a median of 20 mm and an interquartile range (IQR) of 13.25 mm in latero-lateral diameter. Regarding the form of clinical presentation, we observed secondary amenorrhea in the female patient (case 2) with prolactin secreting PA, growth retardation in 2 patients with prolactin-secreting PA (cases 1,3) one of whom also presented galactorrhea, 2 patients with gigantism with growth hormone secreting PA (cases 4,5), one with headache with non-producing PA (case 6) and another patient with symptoms of polyuria and primary polydipsia (3L/day) with FSH secreting PA (case 7) (Table 1).

At diagnosis, apart from the axis causing the hyperfunction, we observed signs and/or symptoms of endocrine dysfunction of other axes: three patients with prolactin-secreting PA and FSH-secreting PA (cases 1,3,7) presented delayed or arrested puberty with decreased testosterone values ​​for their pubertal stage: testosterone 34 ng/dl, 154 ng/ml and 10 ng/dl (nv:300 ± 22 ng/dl). Two patients with prolactin-secreting PA (cases 1,3) had growth retardation (growth rate below -2SDS last year, length -2.5 SDS and -1.68 SDS, with insulin-like growth factor 1 (IGF1) values ​​low limit for their age (154 ng/ml -1.1SDS and 181 ng/ml -0.6SDS). In addition, patient 7 showed hypothyroidism and ACTH deficiency: cortisol: < 5ug/dl (nv: 16.3 ± 6ug/dl), TSH < 0.1U/l (nv: 1.9 ± 1.28U/l), requiring hormone replacement treatment (Table 1).

Three cases (42%) presented with visual field involvement despite not being the reason for consultation but none of them reported visual symptoms (Table 1). In relation to the radiological study, 3 presented suprasellar extension of the lesion and in patient 5 a giant adenoma (40 mm) was identified (Table 1).

A total of 6 patients (85%) had functioning macroadenomas: 3 patients (cases 1,2,3) presented prolactinomas with mean prolactin level of 551 ng/ml and macroprolactin was ruled out, and 2 patients (cases 4,5) with somatropinomas with IGF1 levels + 3SDS and + 5SDS respectively. Patient 7 had an FSH-secreting macroadenoma with a value 3 times above the upper limit of the adjusted value for Tanner's stage and sex of the patient together with multiple hormonal deficiencies: testosterone: 10 ng/dl (nv:300 ± 22 mg/dl), cortisol: < 5ug/dl (nv: 16.3 ± 6ug/dl), TSH < 0.1U/l (nv: 1.9 ± 1.28U/l). The alpha unit of FSH was not assayed. Patient 6 did not have elevated hormones or deficiency, which is why the case was classified as a non-functioning macroadenoma (Table 1).

Regarding treatment, all prolactinomas were treated with cabergoline with weekly doses initially 0.5 mg/week for two weeks and after good tolerance it was progressively increased until 1 mg/week. After three months of follow up cases 2 and 3 achieved the normalization of prolactinemia and reduction of tumor diameter (Table 1). Patient 2 after two years the cabergoline dose could be reduced again to 0.5 mg/week due to normal prolactinemia and stability of the lesion (4 mm). Patient 1 the cabergoline dose was progressively increased until 3 mg/week as after one-month prolactin levels were still elevated (200 ng/ml). The evaluation three month later, patient 1 presented lower prolactin levels (116 ng/dl) and 50% reduction of the lesion (9 mm). Echocardiography was performed in all patients treated with cabergoline and resulted in normal. Patients with non-functioning, GH or FSH-producing macroadenomas were treated with transsphenoidal surgery, obtaining complete resection of the tumor lesion in patient 4 and requiring complementary treatment with subsequent radiotherapy in patients 5, 6 and 7 dues to suprasellar extension of the lesion and invasion of the corpus cavernosum (Table 1).

Regarding the clinical response to treatment, patients with prolactinomas (cases 1, 2 and 3) treated with cabergoline presented with decreased prolactin levels and reduction of the lesion by MRI after three months. Patient 1 after one-month treatment the galactorrhea stopped. Patient 2 with symptoms of secondary amenorrhea resolved a few months after starting cabergoline with regular menstrual cycles and with a control MRI showing a reduction in tumor diameter from 29 to 4 mm. Patients 1 and 3 we will see in the following control appointments how growth and hormonal deficits caused by tumor compression of the pituitary evolve. The rest of the patients who underwent surgery include patient 4 who presented without tumor remnants in a control MRI, with normal IGF1 values ​​and normal final height and complete pubertal development, but with an isolated ACTH deficiency secondary to surgery requiring replacement treatment. The patients who underwent surgery and radiotherapy (cases 5,6,7) presented secondary panhypopituitarism requiring hormone replacement treatment and with remaining tumor remnants in the MRI (Table 1).

Referring to the anatomical pathology of the 4 patients who underwent surgical removal, patient 4 had hormonal immunohistochemical expression and transcription factors compatible with sparsely granulated somatotrophic adenoma, with a prolactin expression focus. A somatotrophic pituitary adenoma was confirmed in patient 5, patient 6 presented adenoma with negative immunoreactivity to all pituitary hormones and patient 7 had an adenoma with immunoreactivity to FSH with a 10–20% cell proliferation index and was p53 negative. After reintervention on patient 7 the anatomical pathology remained the same (Table 1).

A genetic study was carried out in 5 patients using Sanger sequencing directed at the candidate gene. Two patients presented the same pathogenic variant in the *AIP* gene in heterozygosity c.811C > T;pArg271Trp [15]. Following the diagnosis in patient 4, the mother was studied who presented the same pathogenic variant but without clinical manifestations, the paternal grandmother and paternal uncle were shown to be unaffected, and studies in the maternal grandparents are pending. The father was already deceased. Patient 5 had a family history of a maternal aunt and cousin affected by pituitary adenomas with the same pathogenic variant. With these findings and antecedents, the study was expanded to the mother and younger brother who were positive in genetics, but were without clinical manifestations at the time, requiring annual follow-up. Patient 2 subsequently presented a parathyroid adenoma, for which a study of the *MEN1* gene was performed, showing a heterozygous pathogenic variant c.249_252delGTCT;p(IIe85Serfs*33) [16] also present in the sister with a parathyroid adenoma. The parents of the patients could not be studied. Patient 3 resulted in negative for the candidate genes selected (*NF1, AIP, CDKN1B, CDC73, MEN1, RET, PRKAR1A, BRCA1, BECA2, MLH1, MSH2, MSH6).* Patient 1 is more recently diagnosed, and the genetic study is ongoing, while cases 6 and 7 are very old and genetic studies were not performed at the time.

## Discussion

In our series, a higher prevalence of macroadenomas in the male gender is notable. This is consistent with other pediatric series and the wider literature, since pituitary adenomas are more prevalent in women, but macroadenomas in particular predominate in men in childhood and adolescence [1, 3, 8, 9]. We observed in our series that prolactinoma is the most frequent followed by GH-secreting tumor. This has also already been observed in other series such as Menis et al. Barzaghi et al. and Molitch et al. [1, 3, 5]. Although in Jayant et al. and Zhu et al. series prolactinoma is the third most common type, this could be a mere bias as prolactinomas can be managed in primary care facilities with medical treatment [7, 9]. In our series, prolactinoma is the most frequent in all ages, being observed in 42% of all pituitary adenomas (cases 1,2,3). We highlight the presence of 2 very rare tumors in childhood, specifically non-secreting and FSH-secreting, whereas in the wider literature these two types amount to 3–6% of all adenomas observed [3, 8, 9].

With reference to the symptoms, growth arrest predominated in male patients with prolactinomas together with galactorrhea in one case and secondary amenorrhea in the female patient. The clinical manifestations in prolactinomas may differ depending on whether they are prepubertal or pubertal. Headache, visual alteration, growth arrest and primary amenorrhea are more commonly observed prior to puberty and secondary amenorrhea or galactorrhea from puberty [3, 7–10]. In fact, growth arrest is one of the guiding symptoms, as was the case of our series, although in many series it is not one of the most frequent; for example, in Colao et al. [8] it is only presented by a patient with microprolactinoma and Cannavò et al. [17] report only 3 patients out of 30 with delayed growth with micro- or macroprolactinomas. Jayant et al. [9]. Presents headache as the most frequent symptom along with impaired sexual development. It should be noted that frequently in reassessments adolescents with hyperprolactinemia reported experiencing galactorrhea [8], as was the case of our patient 1 who confirmed the presence of galactorrhea for two years after diagnosis.

In relation to the clinical manifestations observed in GH-producing adenomas, hypergrowth is the most frequent, an in fact 95% of patients with GH-producing adenomas present gigantism [3, 5, 9, 10, 13, 18]. Excess GH secretion can cause some systemic complications such as diabetes mellitus, arterial hypertension, sleep apnea, carpal tunnel syndrome, growth of hands and feet, and facial phenotype changes such as prognathism, macroglossia, and prominent forehead [5, 10, 13, 18]. Our patients with somatotropinomas all had gigantism. In contrast, the patient with a non-functioning adenoma only presented with headache with no other clinical or pituitary hormone abnormalities, and the patient with an FSH-secreting adenoma presented symptoms of vasopressin deficiency together with central hypothyroidism, cortisol, and testosterone deficiency. In the literature, gonadotropin-producing adenomas are classified as non-producing and, like them, most patients present with mass effect symptoms such as headache, visual defects, and hypopituitarism [3, 5, 9]. Although in some cases they can be diagnosed incidentally by MRI, Molich et al. [5] argues that there may be a connection between the incidentaloma and the symptoms that cause the MRI to be performed.

In reference to the pathology, this was consistent with the subtype of adenoma initially suspected through hormonal analysis. It should be noted that only patient 4 presented hormonal immunohistochemical expression and transcription factors compatible with sparsely granulated somatotrophic adenoma, with a prolactin expression focus, which was classified according to Mete et al. [19] as somatotropinoma.

Regarding treatment, patient 3 presented maximum prolactin levels of up to 70ng/ml, somewhat lower than those expected for a macroprolactinoma. It is questionable whether it was a non-functioning adenoma with pituitary stalk compression and with decreased negative regulation of prolactin and compression of the rest of the hormonal axis. Even so, it was considered and treated as a prolactinoma since the prolactin levels had been increasing compared to the previous tests and the MRI did not indicate pituitary stalk compression. In addition, the patient responded to medical treatment with cabergoline, reducing prolactin and with normalization of testosterone and the patient's growth; moreover, the control MRI 3 months after treatment showed no increase in tumor size. Furthermore, in the literature Menis et al. [1] and also Colao et al. [8] describe some patients with prolactinomas who presented with serum prolactin between 50-100ng/ml.

Medical treatment with dopamine antagonists is the first choice for prolactinomas, with a response of almost 100% reduction in levels and 90% of tumor mass [3, 14]. Response to medical treatment is considered satisfactory when there is normalization of prolactin levels together with a 50% reduction in tumor size within approximately the first 6 months of treatment [3, 14].

In relation to GH-secreting tumors, the first choice of treatment is surgery, as is the case for non-functioning or gonadotropin-secreting tumors [3, 5, 6, 18]. In our series, these patients all underwent surgery and those with suprasellar extension and invasion of the cavernous sinus also underwent radiotherapy due to the aggressiveness of the lesion. Second-line treatments such as somatostatin analogues were not administered as efficacy depends on the expression of tumor receptors [2, 18]. Furthermore, they are usually indicated in case of persistent or recurrent disease after surgery or when this is contraindicated or not feasible [10, 18]. All the patients undergoing radiotherapy presented panhypopituitarism requiring replacement hormone treatment, while in literature this is observed in 25% of patients undergoing this treatment, especially if the dose is greater than 8-10 Gy [10, 11, 14].

In the adolescent population adenomas present greater aggressiveness since in 5% there is some association with family syndromes such as Familial Isolated Pituitary Adenomas (FIPA), with *MEN1* being among the most frequent, and the other 95% present pathogenic variants to somatic lines of the GNAS gene [2, 9, 13]. Therefore, it is important to perform genetic studies in the form of panels or suspected single gene sequencing in patients with pituitary adenomas under 18 years of age or in patients under 30 with macroadenomas, even if there is no previous family history [9, 12, 13]. In our patients, we observed two familial associations of the mutated *AIP* gene and another with *MEN1*, further to which family studies were carried out, detecting other affected relatives, and enabling their corresponding follow-up and the detection of secondary neoplasms.

## Conclusion

Pediatric pituitary macroadenomas present very different clinical characteristics from adults, since they mostly affect males, are functionally active, and have deleterious effects on the progression of growth and pubertal development, as well as having neuro-ophthalmological manifestations. It is important to carry out genetic studies in patients under 18 years of age due to the higher frequency of genetic and syndromic associations. Once the genetic variant is identified, better monitoring and treatment can be carried out and the family study can be guided.

## Data Availability

The datasets used during this study are available from the corresponding author on reasonable request.

## Abbreviations

PPAs: Pituitary adenomas
MRI: Magnetic resonance
PRL: Prolactin hormone
ACTH: Adrenocorticotropic hormone
GH: Growth hormone
TSH: Thyroid-stimulating hormone
FSH: Follicle-stimulating hormone
LH: Luteinizing hormone
IQR: Interquartile range
IGF1: Insulin-like growth factor 1
FIPA: Familial Isolated Pituitary Adenomas
SSA: Somatostatin analogues
